# Re-examining poikilocytosis in goats: prevalence, type and association with age and disease

**DOI:** 10.3389/fvets.2023.1234233

**Published:** 2023-08-17

**Authors:** Demitria M. Vasilatis, Mary M. Christopher

**Affiliations:** ^1^William R. Pritchard Veterinary Medical Teaching Hospital, School of Veterinary Medicine, University of California, Davis, Davis, CA, United States; ^2^Department of Pathology, Microbiology and Immunology, School of Veterinary Medicine, University of California, Davis, Davis, CA, United States

**Keywords:** caprine, disease, erythrocyte, goat, poikilocytosis, red blood cell

## Abstract

**Background:**

Domestic goats (*Capra aegagrus hircus*) are a food, fiber and companion animal. Abnormal erythrocyte shapes (poikilocytes) are considered normal in young goats, but their association with disease is not well described. Likewise, there is little information on the significance of poikilocytosis in adult goats.

**Objective:**

The objective of this study was to investigate the prevalence, severity and type of poikilocytosis in young and adult goats and its association with age, sex, breed, laboratory results, and underlying disease.

**Methods:**

We retrospectively examined clinical and laboratory data from 1254 goats presented at the University of California-Davis Veterinary Medical Teaching Hospital from 1997 to 2019. We analyzed 313 blood smears from goats with moderate or marked (MOD-MKD) poikilocytosis on initial blood smear evaluation. Number and type of poikilocytes per 1000 red blood cells (RBCs) were enumerated. Laboratory values and primary disease categories were compared with the severity and type of poikilocytosis.

**Results:**

Kids (<6 mos) and juveniles (>6 mos to <1 year) had a higher prevalence of MOD-MKD poikilocytosis (95/210, 45.2% kids; 27/59, 45.8% juveniles) than adult goats (≥1 year; 190/982, 19.3%) (*p* < 0.001). Kids had a higher percentage of elliptocytes, dacryocytes, and schistocytes and a lower percentage of polygonal and spiculated RBCs than juvenile and adult goats (*p* < 0.001). Of goats with MOD-MKD (*vs* NONE-SLIGHT) poikilocytosis, kids had lower HGB and MCH, and higher RDW (*p* ≤ 0.02); juveniles and adult goats had lower HCT, MCV, MCH, and albumin concentration (*p* ≤ 0.01), and all age groups had lower total CO2 concentration and higher PLT counts (*p* < 0.03). Adult goats with MOD-MKD poikilocytosis also had higher BUN:Cr ratios (*p* = 0.005). Gastrointestinal parasitism, Johne’s disease, diarrhea/enteritis, lice, hepatic disease and renal disease (but not urolithiasis) were significantly associated with MOD-MKD poikilocytosis (*p* < 0.001). Goats with hepatic and renal disease had a higher prevalence and percentage of spiculated cells (*p* = 0.001). Goats with Johne’s disease had a higher prevalence of polygonal cells (93.3%) and dacryocytes (66.7%) than other diseases, and elliptocytes predominated in a higher proportion (36.0%) of adult goats with GI parasitism vs other diseases (*p* < 0.05).

**Conclusion:**

These findings suggest that iron deficiency is an important pathophysiologic mechanism of poikilocytosis in juvenile and adult goats, and possibly in kids, whether due to iron-restricted erythropoiesis, chronic hemorrhage, functional iron deficiency, or a combination of these mechanisms. Further investigation into the detection and monitoring of iron deficiency and the value of poikilocytosis as a diagnostic marker of iron status in goats is warranted.

## 1. Introduction

Poikilocytosis is the presence of abnormal erythrocyte shapes in peripheral blood. They are sometimes breed-related and of no clinical significance, such as the fusiform red blood cells (RBCs) observed in Angora goats that result from linear polymerization of hemoglobin (1). However, shape changes in many species often are associated with specific diseases and biochemical changes, and can serve as diagnostic markers as well as result in anemia (2).

Young ruminants develop poikilocytes in the first months of life that are largely attributed to hemoglobin class switching (3–5). At birth, goats have predominantly fetal hemoglobin (Hb F), which is replaced by 80–100% hemoglobin C (Hb C, neonatal hemoglobin) at 40–60 days and subsequently by adult hemoglobin (Hb A) (4, 5). Poikilocytes associated with Hb C occur between birth and 3 months of age and are described primarily as elongated (elliptocytes) and pear-shaped (dacryocytes), with fewer triangular and polygonal cells and occasional schistocytes (3–8). Although poikilocytosis is generally considered physiologic, it is often concurrent with anemia, and several studies in calves have found evidence for iron deficiency (9–11). Nursing animals, especially piglets but also in most domestic species, can develop transient iron deficiency due to high growth rates and the low iron concentration in milk (12). Extreme poikilocytosis has been observed in sick goat kids and calves, but specific disease effects were not investigated (11, 13). Hb C is also the predominant form of hemoglobin synthesized during experimental blood loss in adult goats, which results in poikilocytosis similar to that observed in kids (14). Poikilocytosis has also been reported in clinically healthy juvenile and adult goats, without an apparent cause (2, 7, 8). Little information is known regarding the role of underlying disease, including naturally-occurring blood loss, in the development of poikilocytosis in young and adult goats.

The objectives of this study were to retrospectively characterize the prevalence, severity and type of poikilocytes in a cohort of young and adult goats with naturally-occurring disease and to investigate associations between poikilocytosis and physiologic factors (age, sex, and breed), hematology and biochemical findings, and underlying disease. We hypothesized that poikilocytes in young and adult goats would be associated with specific diseases and differences in laboratory values, including those associated with hemorrhage and/or iron deficiency. The results of this study could provide important insights into underlying pathophysiologic mechanisms that affect the prevalence and type of poikilocytosis in goats.

## 2. Materials and methods

### 2.1. Goat selection and demographic data

We conducted a retrospective, observational cohort study of goats presented to the William R. Pritchard Veterinary Medical Teaching Hospital at the University of California–Davis from January 1997 (the earliest year for which blood smears were available) through December 2018. Electronic medical records were searched for all visits by caprine species with a concurrent complete blood count (CBC) and chemistry panel. For goats with multiple visits, only the first visit with a CBC and chemistry panel was included in the study. Records with missing laboratory reports and herd visits in which individual goats could not be clearly linked with laboratory reports were excluded.

Goat age, sex, and breed were recorded. Goats were categorized as kids (1 day to 6 months), juveniles (>6 months to <1 year), adults (≥1 year to 10 years) or geriatric (>10 years). Sex was categorized as female, male, and castrated male (wether).

### 2.2. Hematology and biochemistry data

Hematology data included red blood cell (RBC) count, hemoglobin concentration (HGB), hematocrit (HCT), mean cell volume (MCV), mean cell hemoglobin (MCH), mean cell hemoglobin concentration (MCHC), red cell distribution width (RDW), reticulocyte count (RETIC), nucleated RBCs (NRBC), total leukocyte count (WBC), differential leukocyte count (neutrophils, immature neutrophils [bands and metamyelocytes], lymphocytes, monocytes, eosinophils, and basophils), platelet (PLT) count, mean platelet volume (MPV), total plasma protein concentration (TPP), and fibrinogen concentration. From January 1997 to September 2001 hematology results were obtained using a Baker Systems 9110 Plus hematology analyzer (BioChem ImmunoSystems Inc., Allentown, PA, United States). From September 2001 to December 2018 results were obtained using an ADVIA 120 hematology analyzer with the goat channel in MultiSpecies System Software (Siemens Healthcare Diagnostics Inc., Tarrytown, NY, United States). Differential counts were obtained automatically by the ADVIA analyzers or manually by counting 200 leukocytes in Wright-Giemsa–stained blood smears. Blood smears also were evaluated for red cell morphology. TPP concentration was determined by refractometry and fibrinogen concentration was determined using the heat precipitation method.

Poikilocytosis was semiquantified in the original CBC report as none, slight, moderate, or marked by the technologist who evaluated the smear. Based on this evaluation, blood smears were retrieved and evaluated for all goats with moderate or marked (MOD-MKD) poikilocytosis. Smears were re-examined by a board-certified clinical pathologist and the percentage (%) of each poikilocyte type (of 1,000 RBCs counted) was determined. The predominant poikilocyte was defined as that with the highest percentage.

Biochemical data included sodium, potassium, chloride, total CO_2_, phosphorus, calcium, urea (BUN), creatinine, glucose, total protein, albumin, and total bilirubin concentrations and sorbitol dehydrogenase (SDH), aspartate transaminase (AST), creatine kinase (CK), alkaline phosphatase (ALP), and gamma-glutamyl transferase (GGT) activities. Anion gap, BUN:creatinine (BUN:Cr) ratio, globulins concentration, and albumin:globulin (A:G) ratio were calculated automatically by the analyzers. Biochemical results were obtained on a Hitachi 717^c^ from 1997 to 2005, a Roche Hitachi 917 from 2006 to 2010, and a Hitachi Cobas c501 analyzer from 2011 to 2018 (Roche Diagnostics Corporation, Indianapolis, IN, United States). When instruments were upgraded, results were calibrated to retain consistency in results between analyzers.

### 2.3. Gastrointestinal parasite data

Results of fecal analyses were recorded when available. Major gastrointestinal (GI) parasites were identified by flotation, sedimentation, and/or McMaster’s tests. Results for *Trichostrongylus sp.* and *Eimeria sp.* (coccidia) were categorized as negative, few, or many/positive (Table 1) (12, 15). Coccidiosis was diagnosed in some cases at necropsy, based on intralesional organisms and associated pathologic findings. *Haemonchus sp.* was identified by fecal larval culture and/or at necropsy. *Cryptosporidia sp.* or *Giardia sp.* were observed in fecal specimens and/or confirmed by fluorescent antibody test.

**Table 1:** Categorization of fecal analysis results for *Trichostrongylus* and *Eimeria spp*.*

Parasite	Negative	Few	Many/Positive
Trichostronglus	<50 epg, 1+, none or rare, ≤10/LPF	50–499 epg, 2+, small #, moderate, observed but not quantified, 10-100/LPF, <10/HPF	≥500 epg, 3+, large #, >100/LPF; >10/ HPF; *Haemonchus* in feces or many at necropsy
Eimeria	<50 opg	50–4,999 opg, <100/HPF	≥5,000 opg and/or necropsy diagnosis of moderate to severe enteritis with intralesional coccidia, >100/HPF

epg = eggs per gram; opg = oocysts per gram; LPF, low power field; HPF, high power field.

### 2.4. Primary disease diagnosis

Final clinical diagnoses (as listed by the clinician in the medical record) and pathology diagnoses (as reported by the pathologist in the necropsy and/or biopsy report) were recorded. Where multiple diseases or lesions were present, the primary disease diagnosis was based on the presenting signs together with the primary clinical/pathologic diagnosis (usually the first listed, or as described in the record or report). Necropsy results were from the same visit at which the laboratory data were obtained, or from a later visit if the results were relevant to the primary disease diagnosis.

### 2.5. Statistical analysis

Data were downloaded into an Excel spreadsheet, then transferred to a statistical program (JMP, version 16.1.0, SAS Institute Inc., Cary, NC, United States). Because % poikilocytes and some laboratory data lacked normal distribution (based on visual observation and Shapiro–Wilk tests), the nonparametric Wilcoxon rank sum test was used to assess data between two groups and the Kruskal-Wallis test for more than two groups. Age (in days, months or years) was reported as mean ± SD. Chi square analysis was used to assess categorical data. Correlations between % poikilocytes and laboratory values were done using Spearman’s (ρ) rank correlation for nonparametric data. Best fit with splining was used to compare MCV and MCH and days of age in young goats. A *p* value <0.05 was considered significant.

## 3. Results

Of 2,381 goat visits in the study period, 761 were excluded because they lacked a concurrent CBC and biochemistry panel. Repeat visits (*n* = 359), goats not uniquely identifiable within a herd (*n* = 3), and records in which the CBC had been cancelled or done elsewhere (*n* = 5) also were excluded. A total of 1,254 goats were included in the dataset. Age, sex, and breed distributions were tabulated (Table 2). Age was not reported for 3 goats. For goats with a specific age or birthdate reported, the mean (± SD) age was 4.5 ± 2.3 years for adults (*n* = 773), 12.2 ± 2.1 years for geriatric goats (*n* = 117), 8.7 ± 1.7 months for juveniles (*n* = 16), and 57.2 ± 55.4 days for kids (*n* = 93). Kids were 1–30 days (*n* = 45), 31–60 days (*n* = 16), 61–90 days (*n* = 11) and 91–180 days (*n* = 21) old.

**Table 2:** Age, sex, and breed distribution of the 1,254 goats in the study.

Variable	*n* (%)
Age	
Kid (1 day to 6 mos)	210 (16.7)
Juvenile (>6 to <12 mos)	59 (4.7)
Adult (1–10 yrs)	863 (68.8)
Geriatric (>10 yrs)	119 (9.5)
Unknown or not reported	3 (0.2)
Sex	
Female	698 (55.7)
Male	243 (19.4)
Male castrated	296 (23.6)
Unknown or not reported	17 (1.4)
Breed	
Pygmy	222 (17.7)
Mixed	221 (17.6)
Boer	156 (12.4)
Nubian	164 (13.1)
Nigerian Dwarf	119 (9.5)
Alpine	96 (7.7)
LaMancha	70 (5.6)
Toggenburg	61 (4.9)
Saanen	32 (2.6)
Oberhasli	21 (1.7)
Angora	20 (1.6)
Other (n < 20)	47 (3.7)
Unknown or not reported	25 (2.0)

### 3.1. Prevalence, severity, and type of poikilocytes

**Figure 1:**
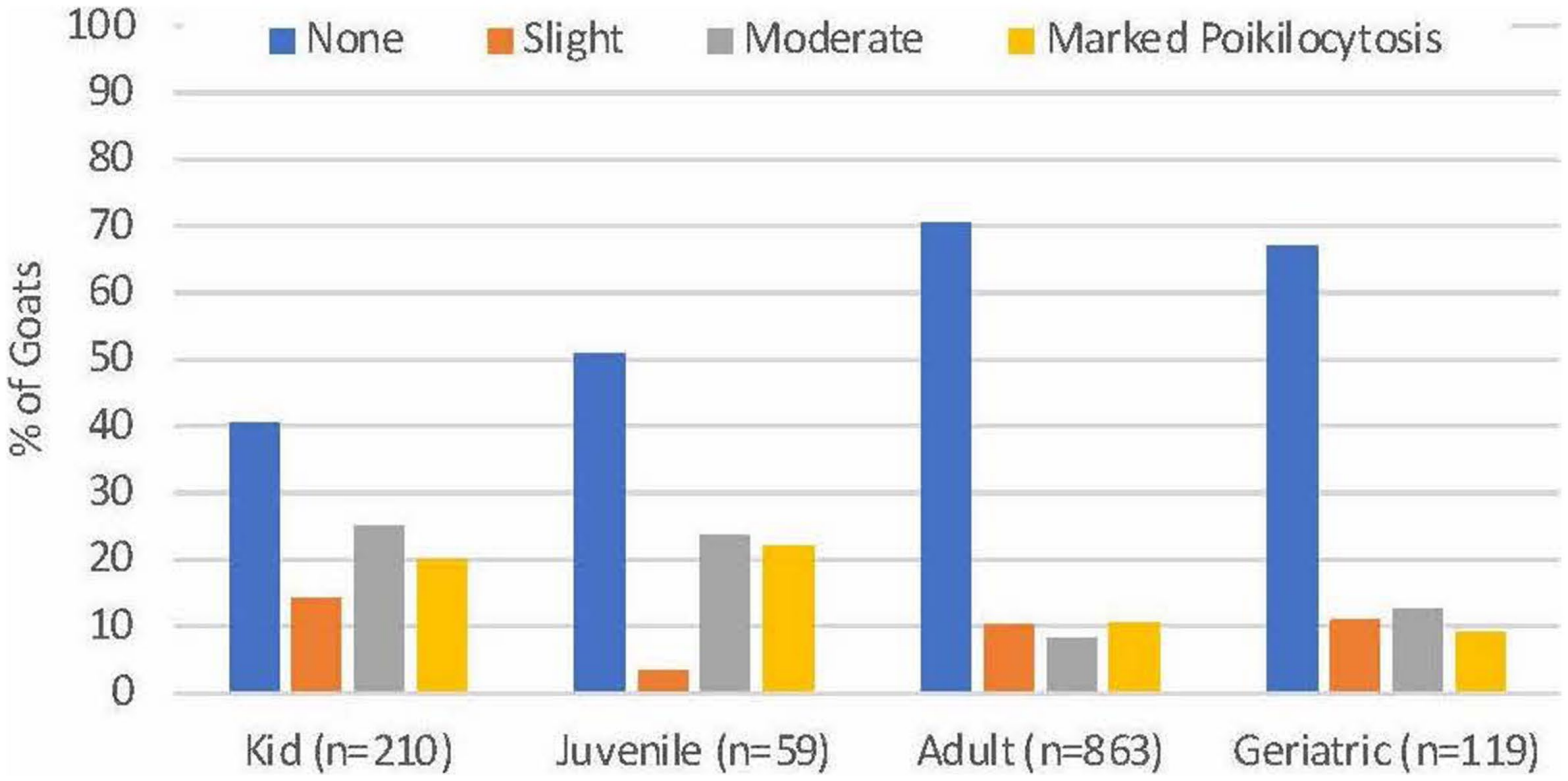
Severity of poikilocytosis in goats of different ages. Significantly higher proportions of kids and juveniles had moderate to marked poikilocytosis compared with adult and geriatric goats (*p* < 0.001).

Of 1,254 goats, 136 (10.8%) had slight poikilocytosis, 155 (12.4%) had moderate poikilocytosis, and 159 (12.7%) had marked poikilocytosis. Poikilocytosis was not reported in 804 (64.1%) goats. Significantly higher proportions of kids (95/210, 45.2%) and juveniles (27/59, 45.8%) had moderate or marked poikilocytosis compared with adults (164/863, 19.0%) and geriatric (26/119, 21.8%) goats (*p* < 0.0001, Chi square, Figure 1). No difference was observed in the severity of poikilocytosis based on sex, within age groups (*p* ≥ 0.11, data not shown). Significantly higher proportions of Angora (13/20, 60.0%) and Boer (67/156, 43.0%) goats and a lower proportion of Nigerian Dwarf goats (13/119, 10.9%) had moderate to marked (MOD-MKD) poikilocytosis compared with other breeds (*p* < 0.001, Chi square). For subsequent analyses, goats having none to slight (NONE-SL, *n* = 939) and MOD-MKD (*n* = 315) poikilocytosis were combined.

**Figure 2:**
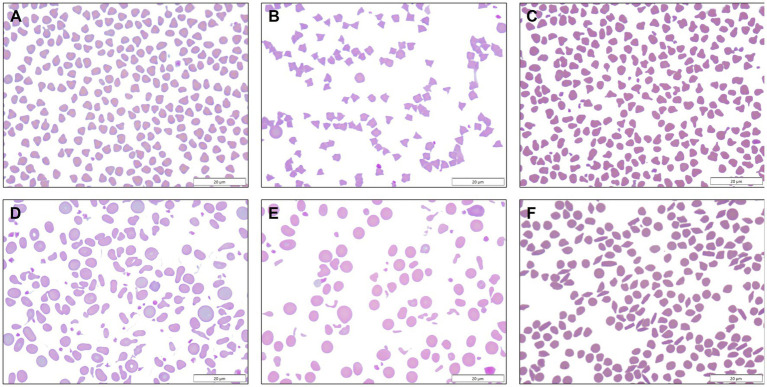
**(A)** Polygonal cells ranged from square to triangular to occasionally diamond or v-shaped, with sharp or rounded corners. **(B)** Spiculated cells comprised a morphologic spectrum from echinocytic to acanthocytic. **(C)** Dacryocytes resembled bowling pins or bicycle seats, and sometimes had long sharp projections. **(D)** Elliptocytes were oval to elongated, occasionally slightly tapered, and were sometimes transversely pinched. **(E)** Schistocytes were small fragments of erythrocytes. **(F)** Fusiform cells were observed in low numbers in Angora goats, sometimes together with matchstick-shaped cells.

**Figure 3:**
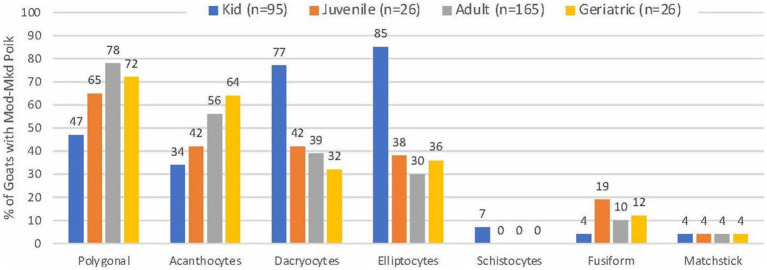
Frequency of poikilocyte types in goats with MOD-MKD poikilocytosis based on age.

**Figure 4:**
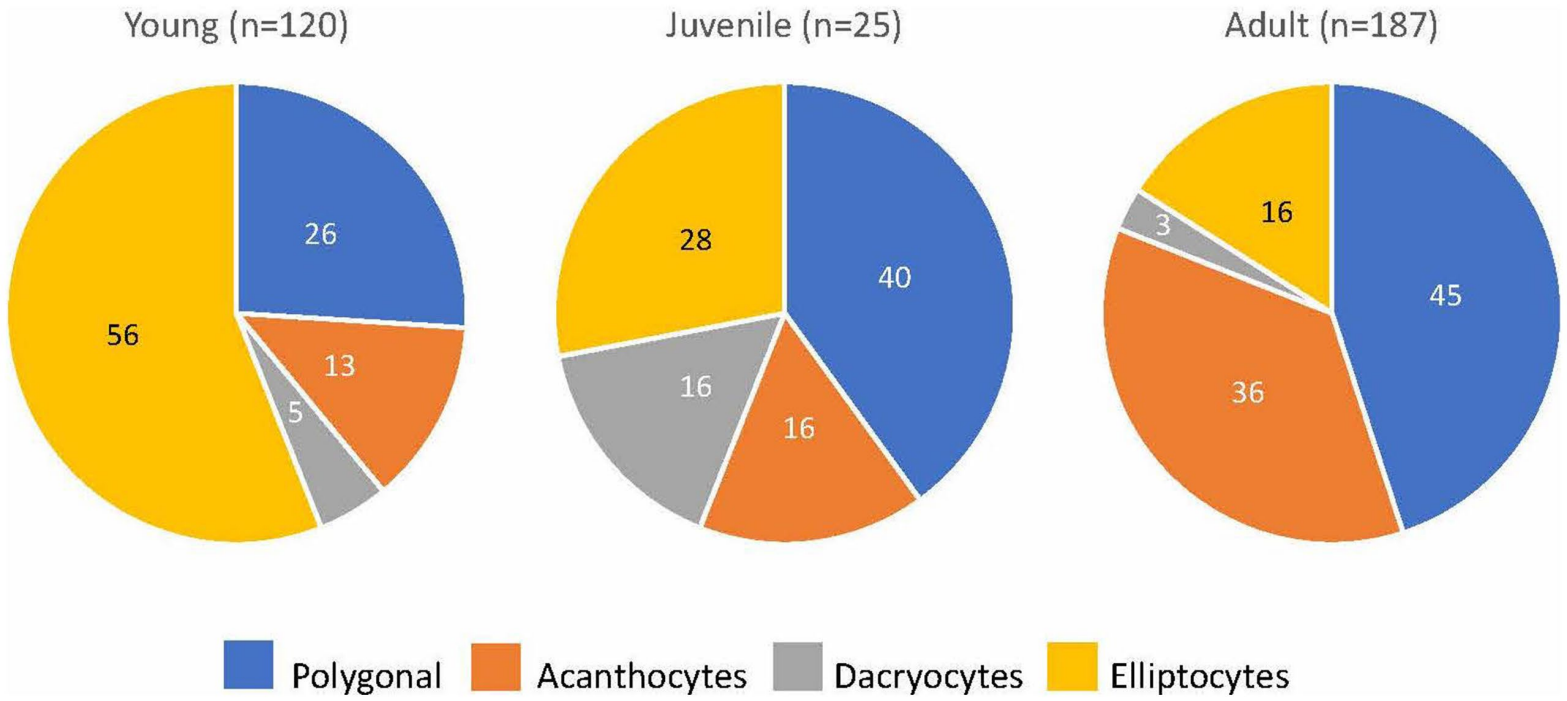
The predominant poikilocyte in goats based on age (% of goats with MOD-MKD poikilocytosis).

Blood smears were available from 313 of 314 goats with MOD-MKD poikilocytosis. Poikilocytes were categorized as polygonal (including triangular), spiculated, dacryocytes, elliptocytes, schistocytes, fusiform, and matchsticks (Figure 2). Elliptocytes and dacryocytes were observed most often in kids, all but 7 of which were < 50 days of age (Figure 3). The percentage of elliptocytes was significantly higher in kids than in other age groups and % dacryocytes was higher in kids compared with adult and geriatric goats (Table 3). Low numbers of schistocytes were observed in 7 kids, 6 of whom were 9–17 days of age. Polygonal cells were the most frequently observed poikilocyte in juvenile, adult, and geriatric goats. The percentage of polygonal cells was significantly lower in kids than in other age groups, and % spiculated cells was lower in kids compared with adult and geriatric goats. Overall, elliptocytes predominated in kids, whereas polygonal cells and acanthocytes predominated in adult goats (*p* < 0.001) (Figure 4). In juvenile goats, the predominant poikilocyte was intermediate between kids and adults, with more elliptocytes/dacryocytes than adults but more polygonal cells than kids. Fusiform cells were found in low numbers and were more prevalent in Angora goats (9/12, 75.0%) compared with other breeds (*p* < 0.01). Because adult and geriatric goats had similar rates of MOD-MKD poikilocytosis and similar types and percentages of poikilocytes, these groups were combined for subsequent analyses.

**Table 3:** Percentage of poikilocyte types in ill young and adult goats with MOD-MKD poikilocytosis.^a^

Poikilocyte Type	Kids (*n* = 95)	Juvenile (*n* = 26)	Adult (*n* = 164)	Geriatric (*n* = 26)	*p*-value
Polygonal (%)	0 (0–70.3)	18.3 (0–55.6)	24.9 (0–77.1)	22.0 (0–62.5)	<0.001
Spiculated (%)	0 (0–48.8)	0 (0–99.6)	11.2 (0–100)	8.3 (0–100)	<0.001
Dacryocytes (%)	7.6 (0–34.4)	0 (0–91.8)	0 (0–48.2)	0 (0–33.6)	<0.001
Elliptocytes (%)	26.0 (0–80.0)	0 (0–57.3)	0 (0–66.9)	0 (0–61.0)	<0.001
Schistocytes (%)	0 (0–21.6)	0 (0)	0 (0)	0 (0)	0.001
Fusiform (%)^b^	0 (0–4.4)	0 (0–77.8)	0 (0–68.8)	0 (0–62.2)	—
Matchsticks (%)	0 (0–2.2)	0 (0–9.0)	0 (0–26.4)	0 (0–14.4)	—

a Data are median (minimum-maximum); Kruskal-Wallis test; — indicates no significant difference.

b Includes Angora goats (5 kids, 5 juveniles, 8 adults, 2 geriatric).

### 3.2. Association of poikilocytosis with hematology and biochemistry data

**Figure 5:**
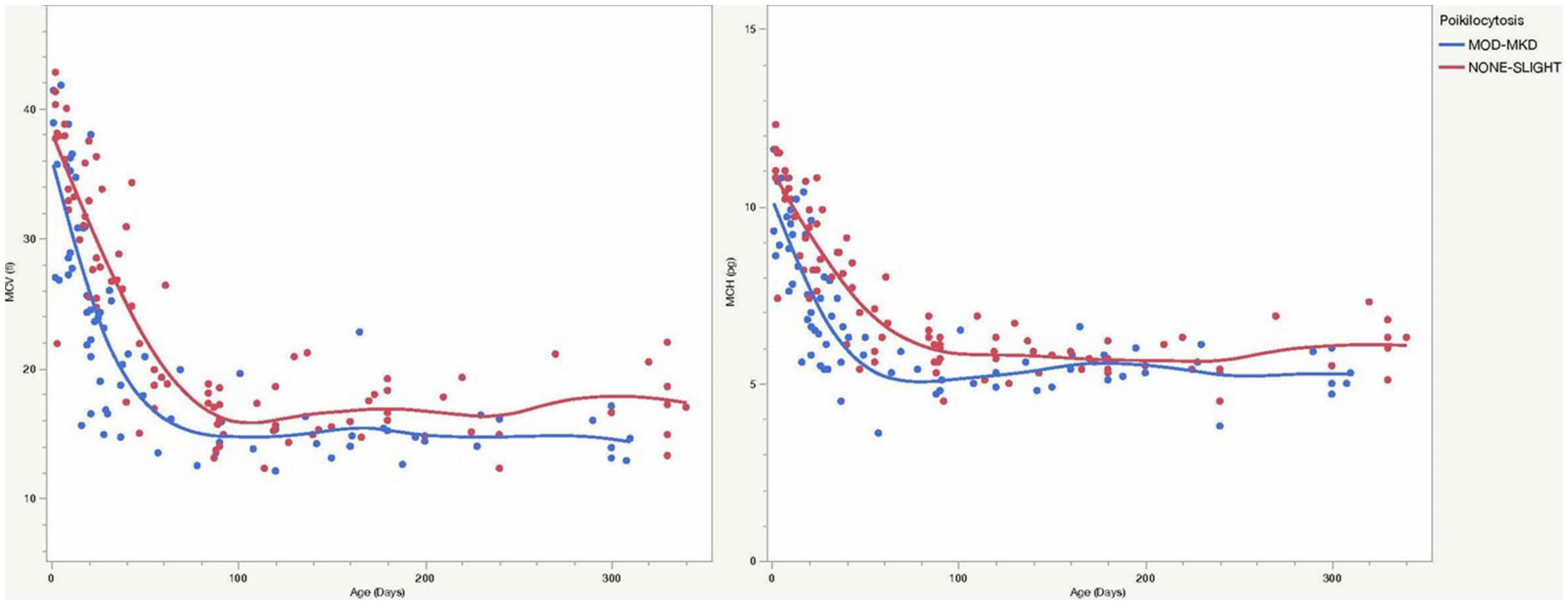
MCV and MCH were consistently lower in young goats with MOD-MKD (*vs* NONE-SL) poikilocytosis, especially between 20 and 45 days of age (*p* < 0.001).

CBC and biochemistry data were available for all 1,254 goats. A few analytes had smaller samples sizes because of the test being optional (RETIC), platelet clumping (PLT, MPV), and differences in test panels (RDW, AST, ALP) (Table 4). Because of significant age differences in multiple analytes (data not shown), laboratory data were analyzed separately for kids, juveniles and adult goats. Both juvenile and adult goats with MOD-MKD poikilocytosis had lower HCT, MCV and MCH than goats having NONE-SL poikilocytosis (Table 4). HGB and MCH were significantly lower in kids with MOD-MKD (*vs* NONE-SL) poikilocytosis. MCV and MCH were slightly but significantly lower in kids with MOD-MKD poikilocytosis throughout the age range, especially between 20 and 45 days of age (Figure 5). All goats with MOD-MKD poikilocytosis had higher PLT counts and lower total CO_2_ concentrations. Eosinophil counts were significantly lower in kids and significantly higher in juvenile and adult goats with MOD-MKD (*vs* NONE-SL) poikilocytosis. Compared with adult goats having NONE-SL poikilocytosis, those with MOD-MKD poikilocytosis had significantly higher neutrophil and monocyte counts; lower sodium, chloride, albumin and total protein concentrations; higher BUN:Cr ratios; and higher SDH, AST, CK, and GGT activities.

**Table 4:** Laboratory results in 1251 goats based on the severity of poikilocytosis.^a^

VARIABLE	KIDS (0–6 MOS)			JUVENILES (>6 to <12 MOS)			ADULTS (≥ 1 YEAR)		
	NONE-SL Poikilocytosis (*n* = 115)	MOD-MKD Poikilocytosis (*n* = 95)	*p*-value	NONE-SL Poikilocytosis (*n* = 32)	MOD-MKD Poikilocytosis (*n* = 27)	*p*-value	NONE-SL Poikilocytosis (*n* = 792)	MOD-MKD Poikilocytosis (*n* = 190)	*p*-value
RBC (X10^6^/μl)	14.9 (5.1–26.5)	14.6 (5.3–27.4)	—	18.1 (7.0–28.9)	18.3 (3.7–24.2)	—	15.1 (3.6–31.4)	13.6 (2.2–26.8)	<0.001
HGB (g/dl)	10.1 (4.7–13.5)	9.1 (3.9–15.4)	0.02	10.5 (4.8–17.4)	9.9 (1.4–13.5)	—	10.2 (2.5–20.4)	8.9 (1.2–17.4)	<0.001
HCT (%)	29.5 (16.5–41.5)	28.9 (13.6–45.1)	—	30.0 (15.4–49.7)	25.4 (4.5–35.9)	0.04	29.6 (7.7–62.8)	25.4 (4.7–47.8)	<0.001
MCV (fl)	19.0 (12.3–45.9)	18.4 (11.8–41.8)	—	16.8 (12.3–24.6)	14.9 (12.3–20.0)	0.001	19.4 (12.5–29.3)	18.6 (11.2–41.3)	0.004
MCH (pg)	6.4 (4.5–12.5)	6.0 (3.6–11.6)	0.006	5.9 (4.5–7.6)	5.3 (3.8–6.2)	0.001	6.7 (4.3–15.1)	6.5 (3.4–13.0)	0.01
MCHC (g/dl)	32.7 (24.6–46.5)	32.5 (24.0–43.3)	—	35.0 (30.5–41.9)	36.6 (30.5–43.5)	—	34.4 (26.4–57.6)	34.8 (25.5–50.0)	0.03
RDW (%)^b^	28.8 (21.7–63.1)	33.8 (21.5–64.4)	0.001	26.3 (22.3–45.6)	25.5 (17.4–42.9)	—	24.2 (18.4–64.7)	24.5 (19.9–46.7)	—
RETIC (/μl)^b^	0 (0–206,090)	0 (0–177,310)	—	0 (0–140,200)	0 (0–66,060)	—	0 (0–252,600)	0 (0–308,560)	0.001
NRBC (/100 WBC)	0 (0–18)	0 (0–9)	—	0 (0–77)	0 (0–57)	—	0 (0–11)	0 (0–15)	0.008
WBC (/μl)	12,740 (2570–52,750)	14,020 (710–54,800)	—	13,975 (1260–67,570)	16,090 (4340–37,620)	—	11,905 (890–61,940)	15,345 (780–64,580)	<0.001
Immature (/μl)	0 (0–3,136)	0 (0–13,769)	—	0 (0–2,465)	0 (0–1859)	—	0 (0–10,484)	0 (0–8,791)	<0.001
Neutrophils (/μl)	7,115 (109–45,893)	7,095 (92–47,676)	—	8,709 (181–58,786)	8,684 (217–28,862)	—	7,872 (70–57,604)	10,230 (122–54,247)	<0.001
Lymphocytes (/μl)	4,284 (1278–13,770)	4,846 (362–24,605)	—	4,767 (378–12,667)	4,343 (1398–13,919)	—	2,769 (305–15,429)	2,855 (428–32,662)	—
Monocytes (/μl)	380 (0–2,923)	399 (7–2,863)	—	334 (0–4,667)	471 (0–1,538)	—	344 (0–5,240)	448 (0–13,675)	0.003
Eosinophils (/μl)	61 (0–572)	0 (0–633)	0.005	0 (0–470)	95 (0–376)	0.03	75 (0–10,002)	41 (0–2,446)	0.02
Basophils (/μl)	74 (0–1,281)	63 (0–632)	—	24 (0–471)	48 (0–396)	—	24 (0–1,206)	1 (0–407)	0.003
PLT (X10^3^/μl)^b^	748 (202–2,415)	1,008 (12–374)	<0.001	532 (16–1928)	780 (16–1729)	0.03	471 (10–1962)	529 (19–1750)	0.001
MPV (fl)^b^	4.7 (3.3–10.8)	4.7 (3.3–0.4)	—	5.7 (4.1–11.9)	4.8 (3.5–8.2)	—	7.1 (3.4–27.8)	6.3 (3.6–16.8)	0.008
TPP (g/dl)	5.9 (3.8–10.0)	5.9 (3.1–9.4)	—	6.3 (4.5–9.4)	6.6 (3.5–8.9)	—	7.2 (3.5–12.3)	6.8 (3.7–11.6)	0.001
Fibrinogen (mg/dl)	300 (100–1,000)	350 (100–1,300)	—	300 (100–1,100)	300 (100–900)	—	400 (100–1,400)	300 (100–1,100)	—
Anion gap (mmol/L)	18 (6–35)	19 (9–42)	—	17 (11–32)	19 (12–46)	—	19 (4–56)	21 (8–64)	<0.001
Sodium (mmol/L)	145 (114–161)	145 (122–208)	—	146 (136–152)	144 (125–158)	—	145 (120–159)	143 (123–185)	<0.001
Potassium (mmol/L)	4.3 (2.5–10.3)	4.2 (2.1–9.8)	—	3.6 (2.0–6.2)	4.1 (2.9–6.8)	0.04	3.9 (1.8–8.9)	3.9 (1.6–8.0)	—
Chloride (mmol/L)	106 (65–128)	106 (79–166)	—	106 (94–134)	107 (81–120)	—	105 (55–123)	104 (69–144)	0.009
Total CO_2_ (mmol/L)	25 (11–54)	24 (6–49)	0.02	26 (0–32)	23 (5–35)	0.03	25 (0–57)	21 (5–53)	<0.001
Phosphorus (mg/dl)	7.5 (1.4–22.1)	7.8 (1.7–18.9)	—	4.9 (1.2–11.5)	6.0 (1.5–23.5)	—	4.3 (0.4–51.7)	5.2 (0.8–37.5)	<0.001
Calcium (mg/dl)	9.7 (5.9–11.7)	9.3 (3.3–19.5)	—	9.3 (5.1–11.4)	8.9 (4.1–17.7)	—	8.9 (2.7–18.5)	8.4 (3.6–15.7)	<0.001
BUN (mg/dl)	16 (2–378)	21 (6–144)	—	23 (9–65)	24 (7–304)	—	20 (4–365)	28 (2–433)	<0.001
Creatinine (mg/dl)	0.5 (0.1–6.9)	0.4 (0.1–5.5)	—	0.8 (0.2–2.3)	0.7 (0.2–16.4)	—	0.9 (0.1–33.0)	1.0 (0.1–26.6)	—
BUN:Cr	32 (3–240)	40 (11–225)	—	28 (10–157)	25 (6–127)	—	21 (2–163)	25 (4–150)	0.005
Glucose (mg/dl)	103 (8–1,006)	100 (1–521)	—	89 (45–263)	95 (25–182)	—	116 (7–882)	115 (0–471)	—
Total protein (g/dl)	5.8 (2.9–9.9)	5.6 (2.9–8.5)	—	6.1 (3.0–9.6)	6.5 (3.3–10.2)	—	7.2 (2.8–13.2)	6.7 (3.5–12.4)	0.001
Albumin (g/dl)	2.5 (0.7–4.3)	2.5 (1.2–3.8)	—	3.0 (1.0–4.0)	2.6 (1.1–4.0)	—	2.9 (0.7–4.5)	2.4 (0.8–4.0)	<0.001
Globulins (g/dl)	2.9 (1.5–7.7)	3.2 (1.2–5.6)	—	2.8 (2.0–7.3)	3.6 (1.7–8.5)	—	4.1 (1.6–11.6)	4.3 (1.9–11.0)	—
A:G	0.85 (0.23–1.95)	0.85 (0.22–1.92)	—	1.03 (0.32–1.60)	0.76 (0.19–1.85)	—	0.71 (0.11–1.95)	0.54 (0.13–1.44)	<0.001
Bilirubin (mg/dl)	0.2 (0–1.6)	0.2 (0.1–16.2)	—	0.2 (0–10.0)	0.2 (0.1–0.8)	—	0.2 (0–4.8)	0.2 (0–5.9)	0.03
SDH (U/L)	25 (4–819)	29 (0–6,040)	0.04	29 (11–1845)	24 (0–385)	—	20 (0–1,596)	27 (0–987)	<0.001
AST (U/L)^b^	92 (0–3,938)	99 (0–16,512)	—	142 (0–2,240)	95 (50–527)	—	99 (0–17,546)	128 (0–4,452)	0.006
CK (U/L)	306 (36–490,644)	350 (59–59,250)	—	213 (64–12,323)	281 (51–1897)	—	236 (0–453,387)	364 (27–50,896)	0.004
ALP (U/L)^b^	227 (0–1,543)	202 (0–2016)	—	133 (0–723)	90 (10–773)	—	59 (0–2,365)	50 (0–479)	—
GGT (U/L)	44 (18–468)	51 (24–677)	0.001	46 (5–423)	45 (26–202)	—	42 (0–925)	45 (0–1720)	0.02

a Data are median (minimum-maximum). Wilcoxon tests were used to compare differences in laboratory data based on severity of poikilocytosis;— indicates no significant difference.

b *n* values are lower for these analytes: RDW (98/70 kids, 28/24 juveniles, 674/167 adults); RETIC (20/23 kids, 7/7 juveniles, 163/78 adults); PLT (108/88 kids, 24/23 juveniles, 504/142 adults); MPV (91/64 kids, 27/22 juveniles, 641/159 adults); AST and ALP (105/91 kids, 26/27 juveniles, 792/177 adults).

In general, polygonal and spiculated cells differed from elliptocytes and dacryocytes in their patterns of correlation with laboratory data (Supplementary Tables S1, S2). In kids, elliptocytes and dacryocytes were negatively correlated with HGB, HCT, MCH, and positively correlated with RDW and PLT (Supplementary Table S1). Polygonal and/or spiculated cells were positively correlated with RBC, HGB, HCT, and MCHC, and negatively correlated with MCV, MCH, RDW and PLT. Spiculated cells were positively correlated with HGB, HCT, BUN, BUN:Cr ratio, anion gap, and phosphorus, and negatively associated with total CO_2_. In adult goats, % elliptocytes and % dacryocytes were negatively correlated with RBC, HGB, HCT, MCV, MCH (Supplementary Table S2) and positively correlated with BUN:Cr. Spiculated cells were positively associated with BUN, creatinine, anion gap, and total bilirubin, and negatively associated with total CO_2_. Juvenile goats had fewer significant correlations: similar to kids, elliptocytes were positively associated with RDW and PLT; similar to adult goats, dacryocytes were negatively correlated with RBC, and spiculated cells were positively correlated with BUN and creatinine.

### 3.3. Association of poikilocytosis with GI parasitism

Gastrointestinal parasites were identified by fecal analysis (*n* = 325) and/or at necropsy (*n* = 17). Positive results (many eggs/oocysts) were found in 36/81 (44.4%) kids, 13/28 (46.4%) juveniles, and 88/230 (38.9%) adults goats and a few eggs/oocysts were found in 14/81 (17.3%) kids, 10/28 (35.7%) juveniles, and 174/230 (75.6%) adult goats. Significantly more adult goats (55/230, 55.9%) were positive for *Trichostrongylus* and *Haemonchus sp.* compared with juveniles (1/26, 3.9%) and kids (3/72, 4.2%) (*p* < 0.001, Chi square). Significantly more kids (24/78, 30.8%) and juveniles (9/28, 32.1%) were positive for *Eimeria sp.* than adult goats (28/230, 12.2%) (*p* < 0.001). More kids (14/75, 18.7%) were positive for protozoa (9 *Cryptosporidium*, 5 *Giardia*) than juveniles (3/26, 11.5%; 3 *Giardia*) and adult goats (5/221, 2.3%; 2 *Cryptosporidium*, 3 *Giardia*) (*p* < 0.001). Thirteen goats were positive for more than one GI parasite.

**Figure 6:**
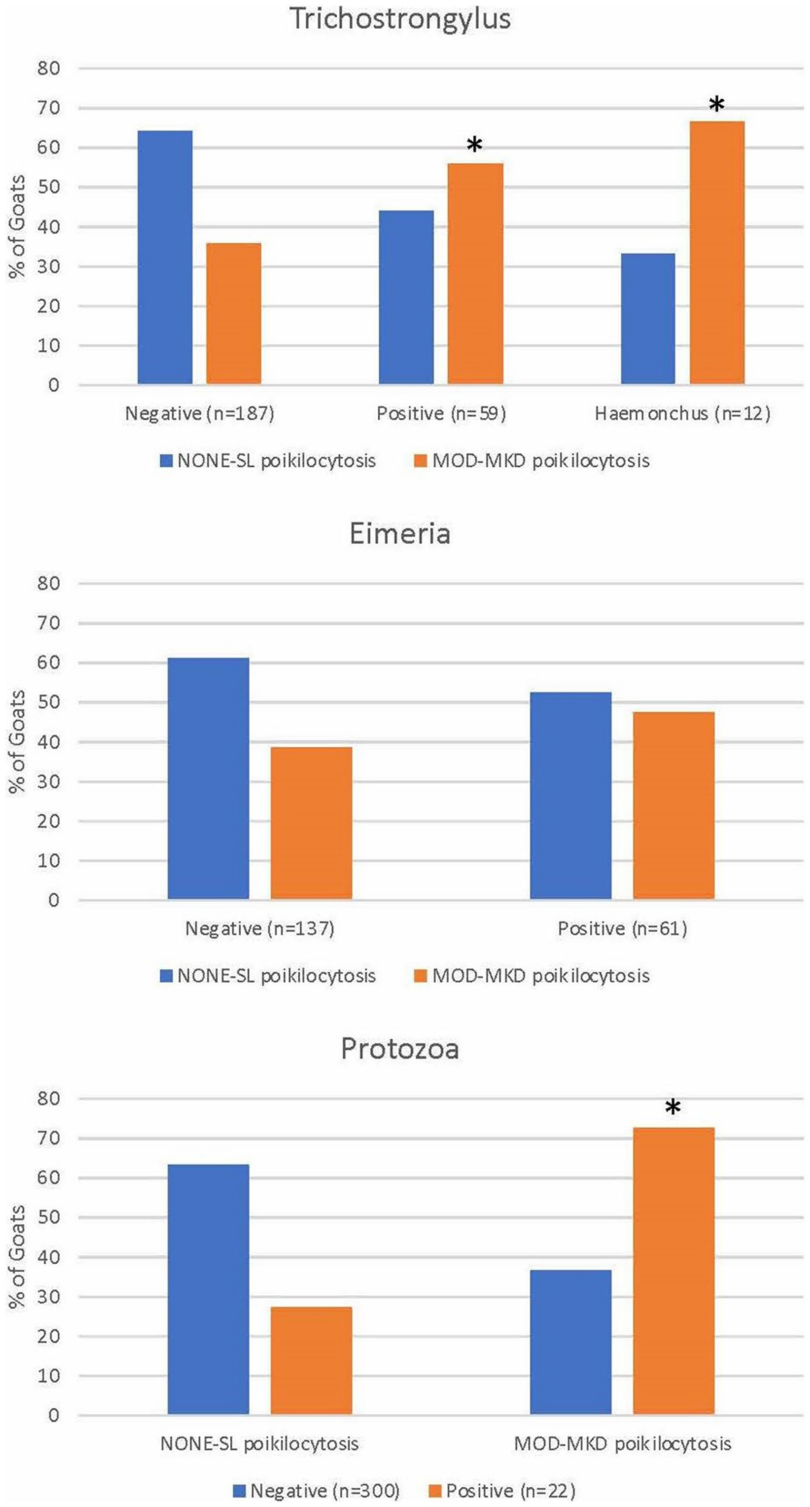
Poikilocytosis in goats with gastrointestinal parasites diagnosed by fecal analysis and/or necropsy. * indicates *p* < 0.05.

A significantly higher proportion of goats positive for trichostrongyles (*p* = 0.013), *Haemonchus sp.* (*p* = 0.013), and protozoa (*p* = 0.001) had MOD-MKD poikilocytosis compared with goats negative for these parasites (Figure 6). Poikilocytosis in goats with few eggs/oocysts was similar to that in negative goats (data not shown). Goats positive for trichostrongyles (primarily adult goats) had a median of 34.2% polygonal cells compared with 12.8% in negative goats (*p* = 0.01, Wilcoxon).

### 3.4. Association of poikilocytosis with primary disease diagnosis

A primary disease diagnosis was determined in 1129/1254 (90.0%) goats, including 210 kids, 59 juveniles, and 982 adult goats. Of these, 418 (36.9%) goats had necropsies confirming the primary diagnosis. Diseased goats were classified into groups based on organ system or pathogenesis. Acute hemorrhage and lice infestation, which were usually secondary to a primary disease, were recorded separately. Clinically healthy goats were blood donors and goats presented for elective procedures. Because of differences in disease prevalence (e.g., neoplasia, reproductive disease) young and adult goats initially were analyzed separately. However, because many diseases (e.g., lice, diarrhea/enteritis, hepatic disease) have similar pathogeneses in young and adult goats, associations of disease with the severity of poikilocytosis also were evaluated for all goats combined. Goats with unknown/open diagnoses (*n* = 125) and diseases with ≤5 cases were not included in statistical analyses.

The strongest associations between disease and MOD-MKD poikilocytosis were for GI parasitism, Johne’s disease, and renal disease (*p* < 0.001) (Table 5). A primary diagnosis of GI parasitism was based primarily on positive fecal analyses, as well as on clinical and necropsy findings. Of 82 goats with a diagnosis of GI parasitism, 80 had fecal analysis or necropsy results; of these, 66 (82.5%) had positive fecal results and/or necropsy confirmation of *Trichostronglus sp.* (*n* = 35, including 12 with *Haemonchus sp*), *Eimeria sp.* (*n* = 25), and/or protozoa (*n* = 15). Although 6/22 goats with Johne’s disease also had GI parasites, exclusion of these goats still resulted in a significant association between Johne’s disease and MOD-MKD poikilocytosis (*χ*^2^ = 6.0, *p* < 0.001). Goats with sucking lice infestation, diarrhea/enteritis, and hepatic disease also had higher proportions of MOD-MKD poikilocytosis than other diseases. Four goats with lice also had GI parasites, 1 had Johne’s disease, and 1 had diarrhea/enteritis; exclusion of these goats still resulted in a significant association between lice and MOD-MKD poikilocytosis (*χ*^2^ = 9.4, *p* < 0.001). The frequency of MOD-MKD poikilocytosis was significantly lower in healthy goats and in goats with neurologic disease, reproductive disorders, and urolithiasis.

**Table 5:** Poikilocytosis in 1129 goats based on primary disease diagnosis.

Primary disease diagnosis	KIDS (0–6 MOS)			JUVENILES (>6 to <12 MOS)			ADULTS (≥ 1 YEAR)			ALL GOATS^c^
	*n*	NONE-SL Poikilocytosis	MOD-MKD Poikilocytosis	*n*	NONE-SL Poikilocytosis	MOD-MKD Poikilocytosis	*n*	NONE-SL Poikilocytosis	MOD-MKD Poikilocytosis	*χ*^2^ *p* < 0.001
Abscess/cellulitis/septicemia	15	6 (40.0)	9 (60.0)	3	2 (66.7)	1 (33.3)	54	46 (85.2)	8 (14.8)	–
Acute hemorrhage^b^	1	1 (100)	0 (0)	2	2 (100)	0 (0)	8	7 (87.5)	1 (12.5)	–
Arthritis	10	3 (30.0)	7 (70.0)	0	—	—	20	15 (75.0)	5 (25.0)	–
Cardiac disease	1	1 (100)	0 (0)	0	—	—	4	2 (50.0)	2 (50.0)	–
Dermatitis	1	0 (0)	1 (100)	0	—	—	11	10 (90.1)	1 (9.1)	–
Diarrhea/enteritis	17	5 (29.4)	12 (70.6)^a^	2	2 (100)	0 (0)	12	9 (75.0)	3 (25.0)	**6.1**
GI disease (other)	12	5 (41.7)	7 (58.3)	1	1 (100)	0 (0)	58	46 (79.3)	12 (20.7)	–
Healthy	5	5 (100)^a^	0 (0)	2	2 (100)	0 (0)	19	19 (100)^a^	0 (0)	**6.7**
Hemolysis	2	1 (50.0)	1 (50.0)	2	2 (100)	0 (0)	13	11 (84.6)	2 (15.4)	–
Hepatic disease	11	4 (36.4)	7 (63.6)	2	2 (100)	0 (0)	21	14 (66.7)	7 (33.3)	**3.1**
GI parasitism	25	11 (44.0)	14 (56.0)	9	1 (11.1)	8 (88.9)^a^	45	20 (44.4)	25 (55.6)^a^	**36.4**
Johne’s disease	0	—	—	0	—	—	22	7 (31.8)	15 (68.2)^a^	**15.2**
Lice infestation^b^	6	2 (33.3)	4 (66.7)^a^	2	0 (0)	2 (100)	12	5 (41.7)	7 (58.3)^a^	**8.4**
Mastitis	0	—	—	0	—	—	55	47 (85.4)	8 (14.5)	–
Metabolic disease	13	8 (61.5)	5 (38.5)	1	0 (0)	1 (100)	29	19 (65.5)	10 (34.5)	–
Myopathy	9	5 (55.6)	4 (44.4)	0	—	—	11	10 (90.9)	1 (9.1)	–
Neoplasia, benign	0	—	—	0	—	—	15	11 (73.3)	4 (26.7)	–
Neoplasia, malignant	0	—	—	0	—	—	56	47 (83.9)	9 (16.1)	–
Neurologic disease	10	9 (90.0)^a^	1 (10.0)	3	2 (66.7)	1 (33.3)	28	27 (96.4)^a^	1 (3.6)	**5.4**
Orthopedic	8	6 (75.0)	2 (25.0)	1	1 (100)	0 (0)	31	26 (83.9)	5 (16.1)	**–**
Peritonitis/pyothorax/pyometra	3	2 (66.7)	1 (33.3)	2	1 (50.0)	1 (50.0)	22	15 (68.2)	7 (31.8)	–
Pneumonia	21	12 (57.1)	9 (47.9)	8	3 (37.5)	5 (62.5)	66	56 (84.8)	10 (15.2)	–
Polioencephalomalacia	5	3 (60.0)	2 (40.0)	5	3 (60.0)	2 (40.0)	20	18 (90.0)	2 (10.0)	–
Renal disease	0	—	—	3	1 (33.3)	2 (66.7)	19	7 (36.8)	12 (63.2)^a^	**12.2**
Reproductive	0	—	—	0	—	—	62	53 (85.5)	9 (14.5)	**3.1**
Toxicosis	1	1 (100)	0 (0)	1	0 (0)	1 (100)	15	14 (93.3)	1 (6.7)	–
Trauma	5	2 (40.0)	3 (60.0)	1	1 (100)	0 (0)	27	23 (85.2)	4 (14.8)	–
Upper respiratory disease	1	0 (0)	1 (100)	0	—	—	5	5 (100)	0 (0)	–
Urolithiasis	9	9 (100)^a^	0 (0)	7	4 (57.1)	3 (42.9)	149	133 (89.3)^a^	16 (10.7)	**13.1**

a Results are significantly different (*p* < 0.05, Chi square) within age group, based on severity of poikilocytosis.

b Secondary to primary disease diagnoses, so analyzed separately in comparison with healthy goats.

c Diseases significantly associated with the severity of poikilocytosis (*χ*^2^ ≥ 3.0) for all goats combined (including 3 goats of unknown age, all of which had GI parasitism). Significantly higher frequency of MOD-MKD poikilocytosis is shown in red; significantly lower frequency of MOD-MKD poikilcytosis is shown in blue.

Laboratory data for the 6 diseases associated with MOD-MKD poikilocytosis were tabulated for all goats combined and in comparison with healthy goats (Table 6). Differences in laboratory data among diseases largely reflected their pathogenesis, including azotemia and electrolyte abnormalities in renal disease, hyperbilirubinemia and enzymopathies in hepatic disease, inflammation and severe hypoproteinemia in Johne’s disease, and eosinophilia in GI parasitism and diarrhea/enteritis. Median BUN/Cr ratio was highest in goats with GI parasitism and lice. Microcytic, hypochromic anemia was most evident in goats with GI parasitism, lice, and Johne’s disease. Median PLT counts were higher in goats with diarrhea/enteritis, GI parasitism, and lice. No significant difference was found in the prevalence or percentage of specific poikilocytes among primary diseases associated with MOD-MKD poikilocytosis in kids. The 4 juvenile goats with a predominance of dacryocytes had GI parasitism. A higher prevalence and % spiculated cells was found in adult goats with hepatic disease (prevalence 85.7%; median 48.4%) and in juvenile (prevalence 100%; median 50.8%) and adult goats (prevalence 83.3%; median 59.7%) with renal disease compared with other diseases (*p* ≤ 0.03). Spiculated cells were the predominant poikilocyte in 85.7% of adult goats with hepatic disease and 83.3% with renal disease. Adult goats with Johne’s disease had a higher prevalence of polygonal cells (93.3%) and dacryocytes (66.7%) compared with other diseases (*p* < 0.05, Chi square). Elliptocytes were the predominant poikilocyte in 36.0% of adult goats with GI parasitism, a significantly higher proportion than in adults with Johne’s disease (26.7%), hepatic disease (6.7%) and renal disease (6.7%) (*p* = 0.01, Chi square).

**Table 6:** Laboratory data (median, range) in healthy goats and goats with diseases associated with MOD-MKD poikilocytosis.

Variable	Healthy (*n* = 26)	Diarrhea/Enteritis (*n* = 31)	GI Parasitism (*n* = 82)^a^	Lice (*n* = 24)	Johne’s disease (*n* = 22)	Hepatic disease (*n* = 34)	Renal disease (*n* = 22)	*p*-value^b^
No. kids/juveniles/adults	5/2/19	17/2/12	25/9/45	8/2/14	0/0/22	11/2/21	0/3/19	
RBC (X10^6^/μl)	15.7 (10.5–22.0)	12.8 (6.8–28.9)	14.5 (3.4–26.5)	13.0 (7.7–20.1)	12.1 (3.6–19.3)	14.8 (2.5–24.4)	12.9 (4.1–23.1)	—
HGB (g/dl)	10.2 (8.8–12.4)	9.3 (4.8–17.4)	8.9 (1.2–12.8)	8.1 (4.1–15.4)	6.7 (2.1–12.8)	9.3 (1.8–16.5)	8.4 (3.2–15.9)	0.003
HCT (%)	29.4 (25.7–37.2)	29.0 (15.4–49.7)	24.9 (4.5–39.6)	23.0 (12.6–42.2)	20.0 (6.1–35.2)	27.1 (6.1–46.5)	23.9 (9.0–42.6)	0.001
MCV (fl)	19.7 (14.9–30.9)	19.6 (12.1–40.3)	17.3 (11.2–36.3)	17.9 (12.4–32.9)	17.0 (12.6–22.7)	18.5 (13.5–35.8)	18.6 (14.7–23.2)	0.04
MCH (pg)	6.8 (5.1–9.1)	6.4 (4.5–11.6)	6.1 (3.4–10.8)	6.2 (4.8–9.4)	6.0 (4.7–7.2)	6.4 (3.6–10.7)	6.6 (4.6–8.3)	0.03
MCHC (mg/dl)	34.1 (29.5–39.7)	33.3 (25.2–42.7)	34.0 (25.5–46.5)	35.4 (28.7–39.2)	35.0 (31.7–41.4)	34.9 (26.7–39.8)	39.5 (30.8–41.1)	—
RDW (%)	23.7 (20.7–30.5)	27.8 (23.1–54.2)	27.0 (19.9–64.4)	26.0 (20.7–44.2)	23.9 (21.6–29.2)	26.7 (20.4–57.9)	26.1 (21.2–42.6)	0.009
RETIC (/μl)	ND	0 (0–190,560)	0 (0–161,600)	0 (0–103,080)	0 (0–26,840)	0 (0–37,450)	0 (0–16,860)	—
WBC (^/^μl)	10,630 (1840–21,320)	14,450 (780–34,490)	16,380 (3040–49,600)	14,080 (7330–54,800)	22,300 (8880–64,580)	13,245 (2530–52,750)	12,685 (4700–30,140)	<0.001
Bands (/μl)	0 (0–370)	0 (0–5,524)	0 (0–6,867)	0 (0–2,983)	0 (0–4,521)	0 (0–3,786)	0 (0–2,646)	0.009
Neutrophils (/μl)	5,756 (276–16,203)	9,393 (148–21,075)	10,815 (904–39,880)	9,005 (2024–47,676)	17,186 (5825–54,247)	8,736 (582–45,893)	9,136 (1867–27,427)	<0.001
Lymphocytes (/μl)	4,232 (1274–7,494)	4,606 (593–9,267)	3,380 (969–24,605)	4,038 (1271–7,923)	3,017 (1986–11,438)	4,238 (1334–12,770)	2,684 (578–9,073)	—
Monocytes (/μl)	288 (74–980)	558 (0–2,457)	422 (0–2,480)	417 (0–1,644)	673 (98–2,498)	414 (42–3,106)	334 (62–1,086)	0.01
Eosinophils (/μl)	138 (0–684)	0 (0–10,002)	66 (0–2,446)	23 (0–462)	19 (0–166)	17 (0–516)	0 (0–359)	0.009
Basophils (/μl)	65 (0–427)	42 (0–285)	28 (0–723)	22 (0–426)	0 (0–285)	20 (0–976)	26 (0–1,206)	0.02
PLT (X10^3^/μl)	469 (154–1,074)	746 (191–1953)	762 (19–2046)	861 (214–1760)	635 (105–1,254)	534 (12–3,736)	569 (161–841)	0.007
MPV (fl)	7.4 (4.2–13.5)	4.9 (4.2–10.3)	5.3 (3.5–12.6)	5.6 (3.5–11.3)	6.5 (4.0–13.3)	5.9 (3.9–14.9)	6.8 (4.0–14.8)	0.001
TPP (g/dl)	7.0 (5.2–9.7)	6.5 (4.0–9.7)	6.2 (3.1–10.0)	6.0 (3.9–9.4)	5.3 (3.7–8.8)	6.5 (−4.0–12.3)	7.1 (4.3–11.0)	<0.001
Fibrinogen (mg/dl)	300 (100–800)	400 (100–1,100)	300 (100–900)	300 (100–800)	300 (100–800)	200 (100–600)	450 (100–1,400)	<0.001
Anion gap (mmol/L)	19 (13–27)	20 (11–41)	18 (11–40)	17 (11–29)	15 (8–32)	21 (9–56)	22 (14–46)	0.004
Sodium (mmol/L)	145 (140–150)	145 (122–208)	144 (114–158)	144 (126–149)	142 (132–151)	146 (136–154)	141 (125–158)	0.002
Potassium (mmol/L)	4.0 (3.0–4.9)	3.8 (2.5–9.8)	4.0 (1.6–6.4)	4.1 (2.5–5.7)	3.6 (2.6–8.0)	3.6 (2.0–6.0)	4.3 (1.9–8.9)	—
Chloride (mmol/L)	106 (100–116)	103 (81–166)	106 (65–119)	104 (81–111)	107 (85–112)	102 (82–114)	97 (59–128)	0.009
Total CO^2^ (mmol/L)	24 (17–35)	23 (12–49)	23 (5–32)	25 (18–35)	22 (16–38)	25 (11–35)	20 (6–36)	—
Calcium (mg/dl)	9.4 (8.0–11.1)	8.7 (3.3–19.5)	8.3 (5.5–15.4)	8.6 (3.6–12.2)	6.9 (5.2–8.0)	8.9 (6.2–16.7)	8.7 (4.8–17.7)	<0.001
Phosphorus (mg/dl)	5.2 (2.1–9.7)	7.2 (1.7–17.4)	5.7 (0.6–15.6)	6.0 (1.6–11.1)	4.9 (1.1–20.8)	6.5 (1.0–12.8)	5.9 (1.3–51.7)	—
BUN (mg/dl)	16 (4–28)	31 (6–144)	23 (6–378)	22 (10–196)	19 (2–99)	27 (9–124)	130 (27–433)	<0.001
Creatinine (mg/dl)	0.6 (0.3–1.6)	0.9 (0.2–5.5)	0.6 (0.1–4.7)	0.4 (0.1–10.7)	0.5 (0.2–1.7)	0.7 (0.1–5.6)	6.9 (0.4–24.8)	<0.001
BUN:Cr	26 (4–46)	31 (12–125)	45 (9–170)	54 (12–220)	34 (4–163)	32 (7–240)	21 (6–157)	0.002
Glucose (mg/dl)	106 (55–242)	121 (18–458)	101 (17–466)	68 (5–284)	84 (15–250)	103 (0–521)	120 (58–781)	—
Total protein (g/dl)	7.0 (4.9–8.3)	6.5 (2.5–9.3)	6.1 (3.1–10.2)	6.3 (3.8–9.6)	5.4 (3.5–8.9)	6.5 (3.6–13.2)	6.4 (2.8–9.6)	0.004
Albumin (g/dl)	3.3 (2.5–4.0)	2.6 (1.1–4.0)	2.2 (0.7–3.8)	2.2 (0.9–3.8)	1.4 (0.9–2.7)	2.5 (1.3–3.7)	2.5 (0.7–3.5)	<0.001
Globulins (g/dl)	3.8 (2.2–5.2)	3.8 (1.8–7.1)	3.9 (1.7–8.5)	3.8 (2.4–6.5)	4.2 (2.3–6.3)	4.0 (2.1–11.6)	4.1 (2.0–6.5)	—
A:G	0.89 (0.60–1.41)	0.77 (0.27–1.56)	0.55 (0.19–1.45)	0.54 (0.25–1.3)	0.37 (0.21–0.63)	0.61 (0.14–1.59)	0.57 (0.31–1.09)	<0.001
Total Bili (mg/dl)	0.2 (0–0-4)	0.2 (0.1–0.8)	0.2 (0–1.2)	0.2 (0–0.5)	0.2 (0.1–1.2)	0.5 (0.1–10.0)	0.2 (0.1–1.1)	<0.001
SDH (U/L)	24 (7–180)	30 (10–144)	26 (0–495)	21 (7–672)	23 (6–528)	110 (6–1,050)	31 (7–206)	0.01
AST (U/L)	71 (55–319)	81 (24–749)	120 (0–1,397)	96 (45–239)	173 (38–781)	302 (0–4,452)	190 (77–709)	<0.001
CK (U/L)	174 (50–2,823)	212 (98–59,250)	338 (65–41,140)	305 (79–18,678)	285 (101–14,464)	373 (85–47,611)	701 (153–9,996)	0.001
ALP (U/L)	139 (30–1,543)	154 (25–598)	63 (0–758)	69 (14–762)	58 (16–209)	70 (0–1,492)	57 (12–773)	0.002
GGT (U/L)	44 (28–73)	54 (21–252)	45 (21–213)	41 (26–227)	62 (28–222)	95 (33–677)	39 (5–202)	<0.001

a Includes 3 goats of unknown age.

b Kruskal-Wallis test; — indicates no significant difference.

## 4. Discussion

The results of this large retrospective study confirm that poikilocytosis characterized primarily by elliptocytes and dacryocytes is a relatively common finding in goat kids, especially in the first 2 months of life. While this coincides with the physiologic timing of hemoglobin class switching, lower HGB and MCH, the correlation of HGB, MCH and HCT with elliptocyte and dacryocyte percentages, and higher PLT counts support the hypothesis that MOD-MKD poikilocytosis in young goats may be associated with iron deficiency, an important cause of poikilocytosis (2, 10, 11).

Iron deficiency anemia due to iron-restricted erythropoiesis can occur in nursing animals during a time of rapid growth, and could be exacerbated by blood loss caused by GI parasitism, diarrhea/enteritis, and lice, diseases associated with poikilocytosis in this study. Lower HCT, microcytic-hypochromic RBCs, and higher PLT counts also were observed in juvenile and adult goats with MOD-MKD poikilocytosis, together with higher BUN:Cr ratios in adults, supporting a similar role for GI blood loss in the development of iron deficiency and subsequent poikilocytosis. In Johne’s disease, also strongly associated with poikilocytosis and microcytic-hypochromic anemia in adult goats, decreased intestinal iron absorption and marked iron sequestration associated with chronic inflammation may limit iron availability. Increased elliptocytes and dacryocytes in adults with GI parasitism and Johne’s disease, and their correlation with erythroid parameters and BUN:Cr ratio, further point toward iron deficiency as a mechanism of RBC shape change. In contrast, spiculated cells in adult goats were strongly associated with renal and hepatic disease, consistent with echinocyte and acanthocyte formation, respectively. Further assessment of iron status in the pathogenesis of poikilocytosis in goats with specific disease conditions is warranted, as is the potential for poikilocytosis to serve as a diagnostic marker of iron deficiency and other disorders in goats.

Age consistently had a more significant effect on RBCs in goats than did sex or breed, as noted previously (8, 13, 16–18). We did not observe sex differences independent of age or disease, and the primary breed difference was the presence of fusiform and matchstick-shaped cells in Angora goats, which were determined previously by electron microscopy to result from polymerization of hemoglobin (1). The majority of Angora goats with MOD-MKD fusiform poikilocytosis were juveniles and adults, consistent with a previous study in which poikilocytosis was mild at birth and gradually became more severe with age (19). This differs from the “physiologic” poikilocytosis in goat kids characterized by elliptocytes and dacryocytes, which is prominent in the first months of life and disappears in transition to adulthood. Fusiform and matchstick cells were seen occasionally in other breeds of goats, such that there may be causes other than genetics for this shape abnormality.

Physiologic changes in caprine RBCs from birth to adulthood complicate the ability to differentiate physiologic from pathologic changes in young goats. Kids have high PCV, MCV and MCH values at birth that decrease in the first month of life, then increase and fluctuate before reaching adult levels by 6–12 months of age (3, 8, 16–18). Reticulocytes, anisocytosis, and NRBCs are also seen in the first month along with “physiologic” poikilocytosis, primarily in the first 2 months. Despite this age-related heterogeneity in RBC values in kids in this study, those with MOD-MKD poikilocytosis had lower HGB and MCH, and higher RDW and PLT counts, findings for which iron deficiency is a primary differential diagnosis. Lower MCH could result from a high proportion of microcytic cells and/or reticulocytes (although RETIC was not significantly higher). Although median MCV was not significantly lower, microcytosis could have been masked by reticulocytes or macrocytes and contributed to the high RDW. Iron deficiency has long been proposed as a cause of poikilocytosis in young ruminants (9–11). Calves <6 weeks old (45 days) had more severe poikilocytosis and significantly lower serum iron, higher total iron binding concentration, and a higher proportion of Hb C than calves >6 weeks old, regardless of health status (10). Iron deficiency was hypothesized by Okabe et al. (11) to cause transient mild anemia, moderate poikilocytosis, and significantly lower serum iron in 1-2-month–old calves, all of which resolved without treatment between days 45 and 90. Lambs develop marked transient anemia in the first month of life, even when iron-supplemented, but a subset developed microcytic RBCs at 15 days of age and were responsive to iron treatment; non-supplemented kids always had lower MCV and MCH values than iron-supplemented kids (20), similar to the consistently lower MCV and MCH values observed over the first 90 days of age in young goats with MOD-MKD poikilocytosis in our study (Figure 5). “Physiologic” iron deficiency in young animals has been attributed to low iron intake during nursing (milk has low iron content) at a time of rapid growth and erythropoietic activity that subsequently exceeds iron availability and may cause anemia (12). Poor placental iron transfer and reduced inte**s**tinal absorption of iron also may be factors (9, 10, 12). Early iron deficiency is most severe in piglets (due to a lack of iron stores) but is a frequent condition in most domestic animal species and in human infants (12, 21). Hypochromic anemia with poikilocytosis in neonatal roan antelope was attributed solely to hemoglobin class switching (e.g., due to a thalassaemia-like imbalance in globin chains); but iron parameters were not evaluated and an outbreak of hemorrhagic calf diarrhea may have altered iron and erythrocyte parameters (6). Although we also did not evaluate iron parameters, our results and those of most other studies in ruminants support iron-restricted erythropoiesis as a contributing factor in the development of physiologic poikilocytosis.

Iron-deficient RBC membranes have decreased deformability and increased oxidative damage of lipids and proteins, which together increase RBC fragility, alter RBC shape, and shorten RBC lifespan via fragmentation and enhanced eryptosis (22–24). Elliptocytes, dacryocytes, keratocytes, and schistocytes are often observed in iron-deficient animals (2). Poikilocytes in iron-deficient calves consisted mainly of acanthocytes and a few schistocytes (10), while antelope calves with hypochromic anemia had primarily elliptocytes (6), with fewer acanthocytes, dacryocytes and schistocytes, as also seen in goat kids in our study. Adult and juvenile goats with GI parasitism and adult goats with Johne’s disease had more elliptocytes and/or dacryocytes than in other diseases, suggesting these poikilocytes may be evidence of underlying iron deficiency. Adult goats with other diseases had primarily polygonal and/or spiculated cells, suggesting other disease mechanisms. Elliptocytes and dacryocytes also are the predominant poikilocytes in humans with iron deficiency and have been found to correlate with the severity of iron-deficiency anemia (25, 26). Dacryocytes are also common in iron-deficient ruminants, including llamas (2), and in ducks with iron-restricted erythropoiesis (27). Although elliptocytes and dacryocytes are not specific to iron deficiency, their concurrence with microcytic and/or hypochromic anemia and thrombocytosis supports a diagnosis of iron deficiency and suggest these poikilocytes could serve as a biomarker.

GI parasites are the most common cause of hemorrhagic anemia in goats (28) and were strongly associated with MOD-MKD poikilocytosis in this study, especially in juveniles and adults. Coccidiosis, caused by *Eimeria sp.*, mainly affects kids 1–6 months of age (7, 15) and was the predominant GI parasite in both kids and juveniles, where it can cause bloody diarrhea, and potentially contribute to iron deficiency and poikilocytosis. The association between fecal oocysts and MOD-MKD poikilocytosis was not statistically significant, however, oocyst numbers do not always correlate directly with clinical disease, and a relatively high level of “physiologic” poikilocytosis due to nursing or other causes may have masked the effect of coccidia. More importantly, a clinical diagnosis of GI parasitism in young goats (~65% of which were positive for *Eimeria* based on fecal analysis) *was* strongly associated with MOD-MKD poikilocytosis. *Cryptosporidia* and *Giardia* infections also were significantly associated with MOD-MKD poikilocytosis in goats in our study, especially kids. These organisms cause severe diarrhea, primarily in neonatal goats up to 4 weeks of age, resulting in poor intestinal absorption and occasionally intestinal hemorrhage (12, 29). *Trichostrongyle* and *Haemonchus spp.*, also strongly associated with poikilocytosis, were primarily observed in adult goats. Heavy burdens of *Trichostrongylus sp.* cause diarrhea and weight loss, with protein-losing enteropathy and malabsorption that decrease iron absorption and increase fecal iron loss (13, 30). Loss of other essential nutrients and impairied iron utilization due to anemia of chronic disease impair erythropoiesis in animals with trichostronglyosis; hypochromasia is sometimes also seen (13). *Haemonchus contortus*, which is hematophagous and lives mainly in the abomasum, is a major cause of GI blood loss and causes significant anemia and hypoalbuminemia (7, 13, 30). *Haemonchus sp.* are not often specifically identified except at necropsy such that their prevalence may have been underestimated in this study. Although heavy infestations of *H. contortous* present with acute blood loss and regenerative (macrocytic-hypochromic) anemia (13, 31), the anemia transitions after 6–14 weeks to nonregenerative iron deficiency anemia that sometimes is microcytic-hypochromic (13). The variable RETIC counts and higher BUN:Cr in goats with GI parasitism (and with hematophagous lice) in our study support the hypothesis that chronic hemorrhage, decreased intestinal absorption, and iron sequestration lead to iron deficiency and subsequent MOD-MKD poikilocytosis in affected goats.

The association between chronic blood loss and poikilocytosis is further supported by a previous study of two adult Angora goats (14). Severe, chronic blood loss anemia, induced experimentally over an 8-week period, resulted in the gradual disappearance of normal discocytes and fusiform cells and the emergence (at ~5 weeks) of a large population of poikilocytes consisting of polygonal/triangular cells, dacryocytes and elliptocytes that persisted for up to 13 weeks. Transient reticulocytosis and a marked increase in HbC also occurred. Thus, chronic hemorrhage (and presumptive iron depletion) in adult goats stimulated an initially regenerative anemia associated with “reverse” hemoglobin switching and poikilocytosis, the latter similar in appearance to that seen in adult goats with GI parasitism in the present study. Neither acute hemorrhage nor hemolytic anemia was associated with MOD-MKD poikilocytosis in our study.

Goats with Johne’s disease develop chronic profuse diarrhea, usually without blood, but with severe abrogation of intestinal absorptive capacity resulting in protein-losing enteropathy and marked hypoalbuminemia (32). Johne’s disease (paratuberculosis) is a granulomatous infection of ruminants caused by *Mycobacterium avian* subsp. *paratuberculosis* (MAP) that primarily affects the intestinal tract. More than other diseases associated with poikilocytosis in this study, Johne’s disease caused an inflammatory leukogram with neutrophilia and monocytosis. And more than other microbial or parasitic diseases, Johne’s disease results in severe anemia of inflammation, with marked iron accumulation in granulomatous lesions and liver, and low serum iron concentration (33, 34). Indeed, MAP is well know for its unique dependence on ferric iron and its unique iron regulatory genes that enable it to sequester large amounts of iron within monocytes and macrophages (35, 36). This massive iron sequestration can result in functional iron deficiency, in which iron is present in high amounts in the body but is unable to be mobilized for erythropoiesis (21). Indeed, goats with Johne’s disease had the lowest HCT, MCV, and MCH values of all the major diseases associated with MOD-MKD poikilocytosis. Thus, we hypothesize that goats with Johne’s disease develop poikilocytosis secondary to severe functional iron deficiency. Functional iron deficiency can occur concurrent with other diseases and other causes of iron deficiency in goats, including GI parasitism, and could interfere with the efficacy of supplemental iron in treating microcytic-hypochromic anemia.

Spiculated cells were significantly overrepresented in goats with renal disease and, to a lesser extent, hepatic disease in both young and adult goats. We used the category of spiculated cells because echinocyte and acanthocyte morphology fell on a continuum that made differentiation difficult; only a few cases were clearly one or the other. The association between MOD-MKD poikilocytosis and renal disease was strong in adult goats and in all goats combined, and spiculated cells predominated in nearly 70% of affected animals. Renal failure, uremia and electrolyte and acid–base abnormalities have been associated with echinocytosis in multiple species (2, 37). Notably, poikilocytosis was not associated with urolithiasis in goats in the present study. Urolithiasis presents acutely and requires rapid intervention, suggesting that a more chronic disease process and/or the biochemical changes associated with renal failure are needed to alter red cell shape. Hepatic disease also was associated with MOD-MKD poikilocytosis and spiculated cells predominated in more than 40% of affected goats. Spiculated cells are consistent with excess membrane cholesterol and altered phospholipids, and are frequently observed in other species with hepatic disease (2). Hepatic disease can also result in functional iron deficiency associated with altered transferrin metabolism, which might explain the predominance of elliptocytes in some goats. The slightly higher activities of SDH and GGT likely reflected the occurrence of hepatic disease in kids and adult goats with MOD-MKD poikilocytosis.

To our knowledge this is the first large study to evaluate naturally-occurring poikilocytosis in goats. Our study does have some limitations. Because of the retrospective design, not all goats had fecal analyses or clinical or pathologic diagnoses. However, the large size of the dataset helped maintain robust sample sizes in most subgroups. In addition, because the study encompassed a long time period, hematology data were obtained on two different analyzers, although a large majority (>80%) of samples were analyzed using the ADVIA 120, which has caprine-specific software and has been favorably evaluated for use in goats (38). We also relied on the initial semiquantitative assessment of poikilocytosis by many different laboratory technologists over the years. A bigger challenge was that many goats had multiple diseases or pathologic findings, and identifying a single or primary problem was sometimes difficult. However, when overlapping diseases raised questions about associations with poikilocytosis (e.g., goats with both Johne’s disease and GI parasites), our initial findings were supported, even after exclusion of animals with both diseases. We used stringent criteria based on generally accepted guidelines for defining a positive fecal result, but as noted earlier, there is not always a direct correlation between fecal egg/oocyst quantity and the severity of infection; goats can shed eggs and oocysts without evidence of disease and parasitized goats may not be shedding or may have received prior anthelminthic treatment. The evaluation of GI parasitism as a primary disease group, which considered clinical as well as laboratory data, may provide a more meaningful assessment of the effects of GI parasites. The study also captured only a single point in time for each goat, such that different disease stages and prior treatment likely contributed to variable laboratory findings, including poikilocytes. Nonetheless, clear patterns emerged, and while not all poikilocytosis could be explained by our findings, valid hypothesies could be developed as to likely pathophysiologic mechanisms that warrant further study.

In conclusion, the laboratory and disease findings in this large dataset of goats suggest that in addition to biochemical changes associated with hepatic and renal disease, iron deficiency may be an important pathophysiologic mechanism of poikilocytosis, whether due to iron-restricted erythropoiesis, chronic hemorrhage and iron loss, functional iron deficiency, or a combination of mechanisms. While the hematologic picture is more complex in kids because of physiological changes in RBCs, the association between iron deficiency and poikilocytosis in adults is strong. The relationships between iron status, the erythroid bone marrow response, HbC synthesis, and the development of poikilocytosis warrant further investigation. Further research also is needed on the detection and monitoring of iron status in goats, including the diagnostic value of poikilocytosis, BUN:Cr ratio, and hepcidin and ferritin assays (39, 40) and differentiation of the mechanisms of iron deficiency. Although none of the healthy goats in this study had more than a few poikilocytes in blood smears, further study is also needed in a larger population of parasite- and disease-free goats to better differentiate physiologic and disease-associated poikilocytosis, especially in kids.

## Data availability statement

The original contributions presented in the study are included in the article/Supplementary material, further inquiries can be directed to the corresponding author.

## Ethics statement

The studies were conducted in accordance with the local legislation and institutional requirements.

## Author contributions

DV and MC contributed to conception and design of the study, generation of figures, and conducted quantitative analyses. MC wrote the first draft of the manuscript and performed statistical analyses, generated all tables, supervised the study and reviewed and edited the manuscript. DV wrote sections of the manuscript. All authors contributed to the article and approved the submitted version.

## Funding

DV was supported by training fellowship NIH T32 OD011147 during preparation of this manuscript.

## Conflict of interest

The authors declare that the research was conducted in the absence of any commercial or financial relationships that could be construed as a potential conflict of interest.

## Publisher’s note

All claims expressed in this article are solely those of the authors and do not necessarily represent those of their affiliated organizations, or those of the publisher, the editors and the reviewers. Any product that may be evaluated in this article, or claim that may be made by its manufacturer, is not guaranteed or endorsed by the publisher.
