# Supplementary material for: Re-examining poikilocytosis in goats: prevalence, type and association with age and disease
Source: Front Vet Sci. 2023 Aug 17;10:1234233. doi: 10.3389/fvets.2023.1234233 (PMC10470038; doi:10.3389/fvets.2023.1234233)
Supplement: Supplementary file 1 [file Table_1.docx]

**Supplemental Table 1**. Correlation between % poikilocyte types and laboratory variables in 95 goat kids.*

| Variable | % Polygonal | % Spiculated | % Dacryocytes | % Elliptocytes | P value |
| --- | --- | --- | --- | --- | --- |
| RBC | .568 | — | — | — | <.001 |
| HGB | .463 | .345 | -.278 | -419 | ≤.006 |
| HCT | .299 | .369 | -.329 | -.461 | ≤.003 |
| MCV | -.378 | — | — | — | <.001 |
| MCH | -.304 | .283 | -.245 | -.274 | ≤.02 |
| MCHC | .434 | — | — | — | <.001 |
| RDW | -.434† | — | — | -.239† | <.05 |
| RETIC | — | — | — | — | — |
| NRBC | — | — | — | — | — |
| WBC | — | — | — | — | — |
| Immature neuts | — | — | — | — | — |
| Neutrophils | — |  | — | — | — |
| Lymphocytes | .215† | — | — | — | .04 |
| Monocytes | — | — | — | — | — |
| Eosinophils | — | — | — | — | — |
| Basophils | — | — | — | — | — |
| Platelets | -.342† | — | — | .262† | ≤.02 |
| MPV | — | — | — | — |  |
| TPP | — | — | — | — | — |
| Fibrinogen | — | — | — | — | — |
| Anion gap | — | .297 | -.376 | -.368 | ≤.004 |
| Sodium | — | — | — | — | — |
| Potassium | — | — | — | — | — |
| Chloride | — | — | — | — | — |
| Total CO2 | — | -.266 | .379 | .349 | ≤.01 |
| Phosphorus | — | .252 | — | — | .01 |
| Calcium | — | — | -.170 | — | .02 |
| BUN | .236 | .483† | -.351 | -.387† | ≤.02 |
| Creatinine | .311 |  | -.207† | -.229† | ≤.05 |
| BUN:Cr | — | -.424 | -.205† | — | ≤.05 |
| Glucose | — | — | — | — | — |
| Total protein | — | — | — | — | — |
| Albumin | — | — | — | — | — |
| Globulins | — | — | — | — | — |
| A:G | — | — | — | — | — |
| Total bilirubin | — | — | — | — | — |
| SDH | — | — | — | — | — |
| AST | — | — | — | — | — |
| CK | — | — | — | — | — |
| ALP | — | — | — | — | — |
| GGT | .304 | — | .211 |  | ≤.04 |

*Spearman’s rank correlation coefficients (ρ); positive correlations are shaded red; negative correlations are shaded blue

†Also in juveniles (n=27)
