# Supplementary material for: Re-examining poikilocytosis in goats: prevalence, type and association with age and disease
Source: Front Vet Sci. 2023 Aug 17;10:1234233. doi: 10.3389/fvets.2023.1234233 (PMC10470038; doi:10.3389/fvets.2023.1234233)
Supplement: Supplementary file 2 [file Table_2.docx]

**Supplemental Table 2**. Correlation between % poikilocyte types and laboratory variables in 190 adult goats.*

| Variable | % Polygonal | % Spiculated | % Dacryocytes | % Elliptocytes | P value |
| --- | --- | --- | --- | --- | --- |
| RBC | .210 | — | -.363† | -.262 | ≤.01 |
| HGB | — | .247 | -.459 | -398 | <.001 |
| HCT | — | .242 | -.453 | -.374 | <.001 |
| MCV | -.199 | .250 | -.252 | -.229 | <.01 |
| MCH | — | .294 | -.334 | -.337 | <.001 |
| MCHC | .227 | — | — | -.206 | <.05 |
| RDW | — | — | — | — | — |
| RETIC | -.344 | — | — | .240 | <.05 |
| NRBC | -.173 | — | .146 | .172 | <.05 |
| WBC | — | — | -.198 | — | .006 |
| Immature neuts | — | — | — | — | — |
| Neutrophils | — |  | -.145 | -.187 | <.05 |
| Lymphocytes | — | — | — | — | — |
| Monocytes | — | .322 | -.162 | -.223 | ≤.02 |
| Eosinophils | — | — | — | — | — |
| Basophils | — | — | — | — | — |
| Platelets | — | -.174† | — | .265† | <.05 |
| MPV | — | — | — | — | — |
| TPP | — | — | -.216 | — | .003 |
| Fibrinogen | .201 | — | -.190 | — | — |
| Anion gap | — | .326 | -.301 | -.315 | ≤.009 |
| Sodium | — | -.182 | — | — | .01 |
| Potassium | — | — | — | -.159 | .03 |
| Chloride | — | -.232 | — | — | .001 |
| Total CO2 | — | -.325 | .238 | .190 | ≤.01 |
| Phosphorus | — | — | -.182 | — | .01 |
| Calcium | — | — | -.170 | — | .02 |
| BUN | .173 | .267† | — | -.173† | ≤.02 |
| Creatinine | .152 | .382† | -.294† | -.262† | <.001 |
| BUN:Cr | — | -.226 | .307 | .176 | ≤.01 |
| Glucose | — | — | -.243† | — | <.001 |
| Total protein | — | — | -.146 | — | .04 |
| Albumin | — | — | -.259 | -.156 | ≤.03 |
| Globulins | — | — | — | — | — |
| A:G | — | — | -.153 | — | .03 |
| Total bilirubin | — | .430 | -.253 | -.317 | ≤.02 |
| SDH | — | — | — | — | — |
| AST | — | .246 | — | -.185 | ≤.01 |
| CK | — | .265 | -.186 | -.246 | ≤.01 |
| ALP | — | — | — | -.339 | <.001 |
| GGT | -— | — | — | — | — |

*Spearman’s rank correlation coefficients (ρ); positive correlations are shaded red; negative correlations are shaded blue

†Also in juveniles (n=27)
